# 2492. Implementation of a Hepatitis C Referral Workflow: A Pilot Program

**DOI:** 10.1093/ofid/ofad500.2110

**Published:** 2023-11-27

**Authors:** Katherine Chun, Gary Lau, Jenica Lee, YoungYoon Ham

**Affiliations:** Oregon Health & Science University, Portland, Oregon; Oregon Health and Science University, Portland, Oregon; UCSF, San Francisco, California; Oregon Health & Science University, Portland, Oregon

## Abstract

**Background:**

Direct-acting antiviral (DAA) treatment for hepatitis C viral infection (HCV) is typically initiated in the outpatient setting. Currently at Oregon Health and Science University (OHSU) hospital, there is no standardized method of connecting hospitalized patients who test positive for HCV with outpatient care for treatment. As a result, patients are infrequently referred. We created and implemented a pharmacist-led workflow to evaluate patients and officially refer admitted patients to outpatient care. The goal is to increase the rate of patients who attend follow-up appointments that address HCV diagnosis and treatment after a hospitalization, and increase the rate of patients who are treated.

**Methods:**

The pharmacist-led workflow was implemented from October through December 2021. Pre- and post-implementation (Pre-I and post-I) data was collected and analyzed. Hepatitis C positive patients were identified and assessed for willingness to be treated. If willing, assessment was completed while inpatient and an official outpatient referral was placed. The data includes patients who attended an appointment (primary outcome) and patients who were prescribed a DAA (secondary outcome).

**Figure:**
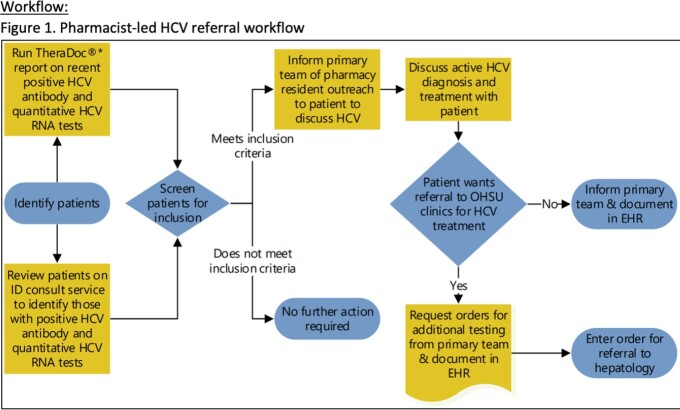

**Results:**

In pre-I, 44 patients were screened and 18 patients met inclusion. In post-I, 75 patients were screened and 33 met inclusion.

Fourteen patients (33%) in post-I and 4 patients (22%) in pre-I were referred to hepatology. Nine patients (50%) in pre-I and 6 patients (14%) in post-I attended a clinic appointment within 3 months. Three patients (17%) in pre-I and 5 patients (12%) in post-I were prescribed a DAA within 3 months.

However, of the referred patients, 2 patients in pre-I and 3 patients in post-I attended an appointment within 3 months. No patients in pre-I and 1 patient in post-I were prescribed a DAA. In both pre- and post-I, 33% of those who attended an appointment received a prescription for a DAA within three months.

**Figure:**
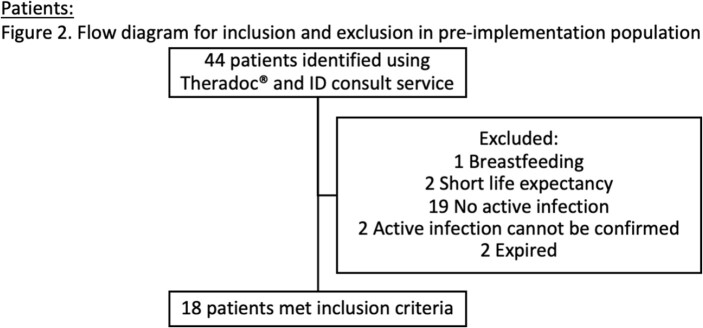

**Figure:**
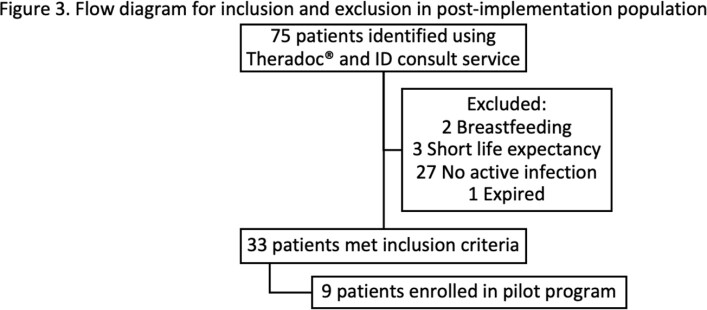

**Figure:**
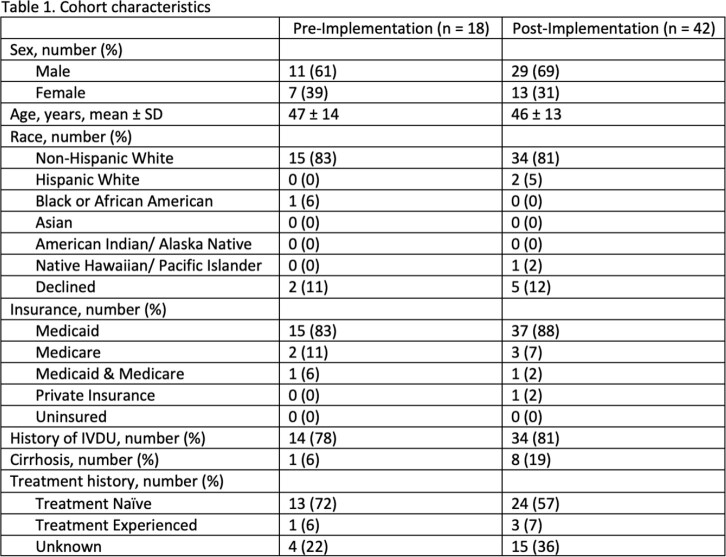

**Conclusion:**

A pharmacist-led HCV referral workflow increased the rate of referrals to hepatology. However, this increase in referrals did not correspond with a substantial increase in appointments or receipt of a prescription.

While the workflow increased referrals, it did not increase patient receipt of DAAs. More intensive interventions may be needed to reach this population.

**Disclosures:**

**All Authors**: No reported disclosures

